# Supplementary figures and images for: Crowdsourcing snake identification with online communities of professional herpetologists and avocational snake enthusiasts
Source: R Soc Open Sci. 2021 Jan 13;8(1):201273. doi: 10.1098/rsos.201273 (PMC7890515; doi:10.1098/rsos.201273)

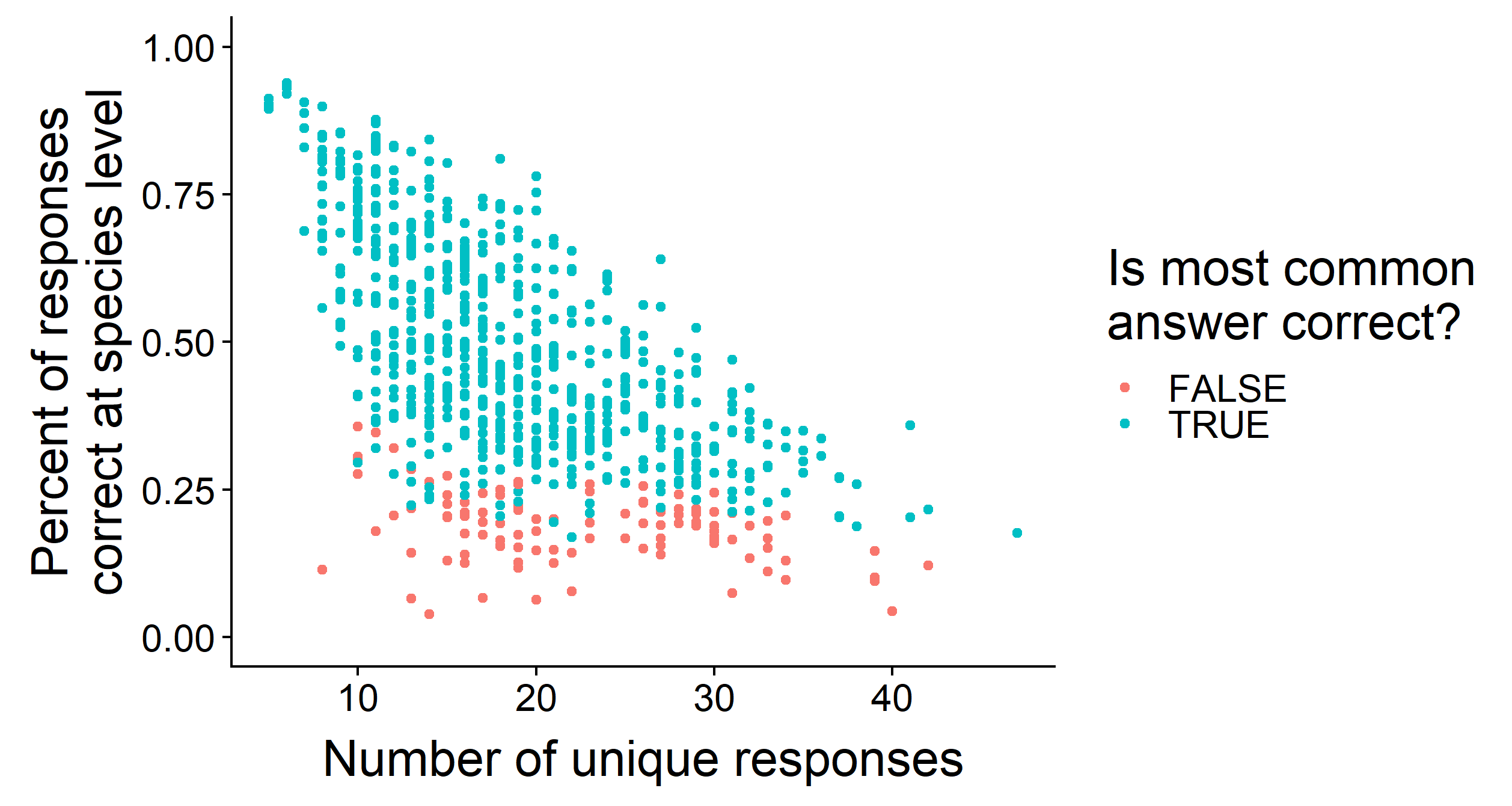

Supplement: Figure S1 [file rsos201273supp1.png]

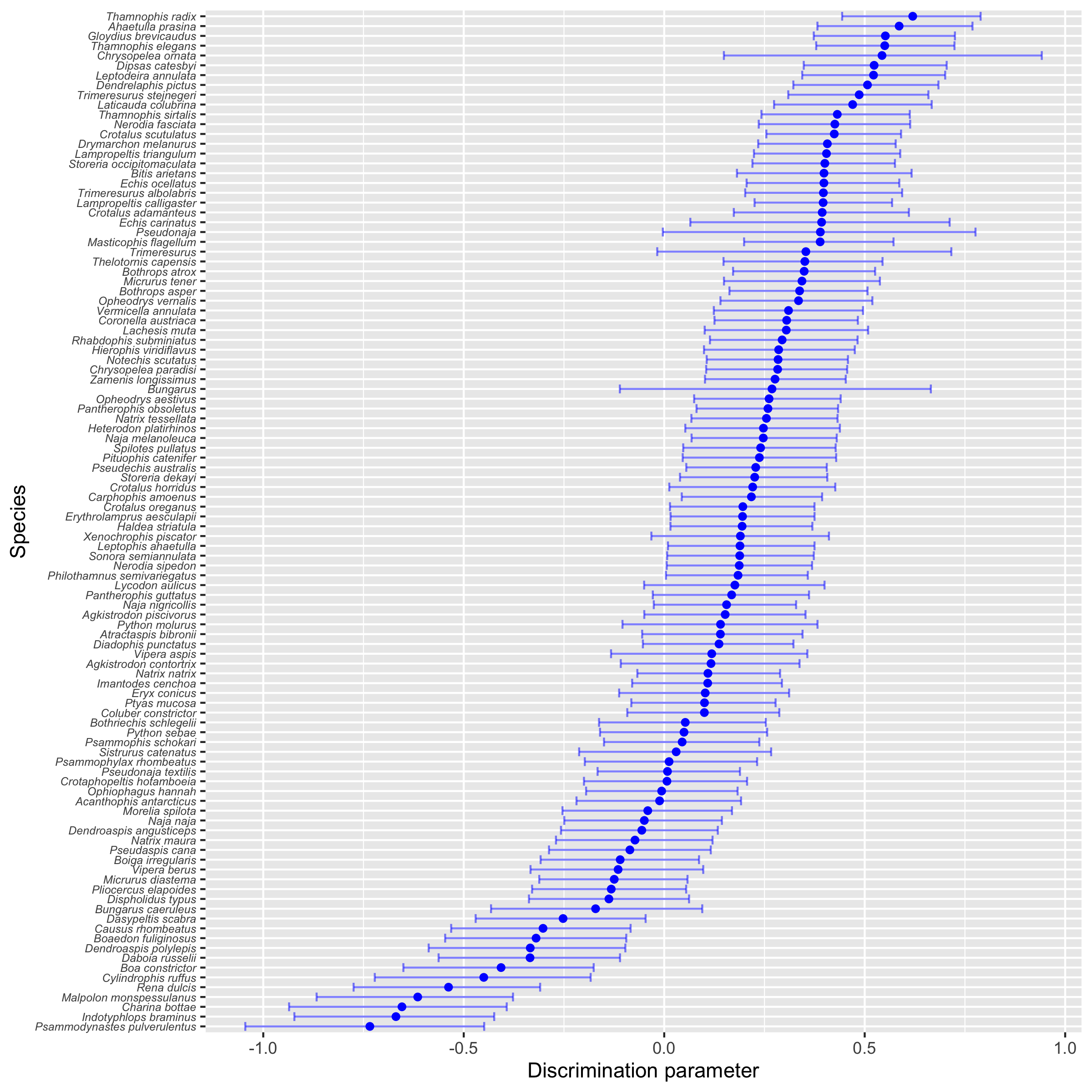

Supplement: Figure S3 [file rsos201273supp2.png]

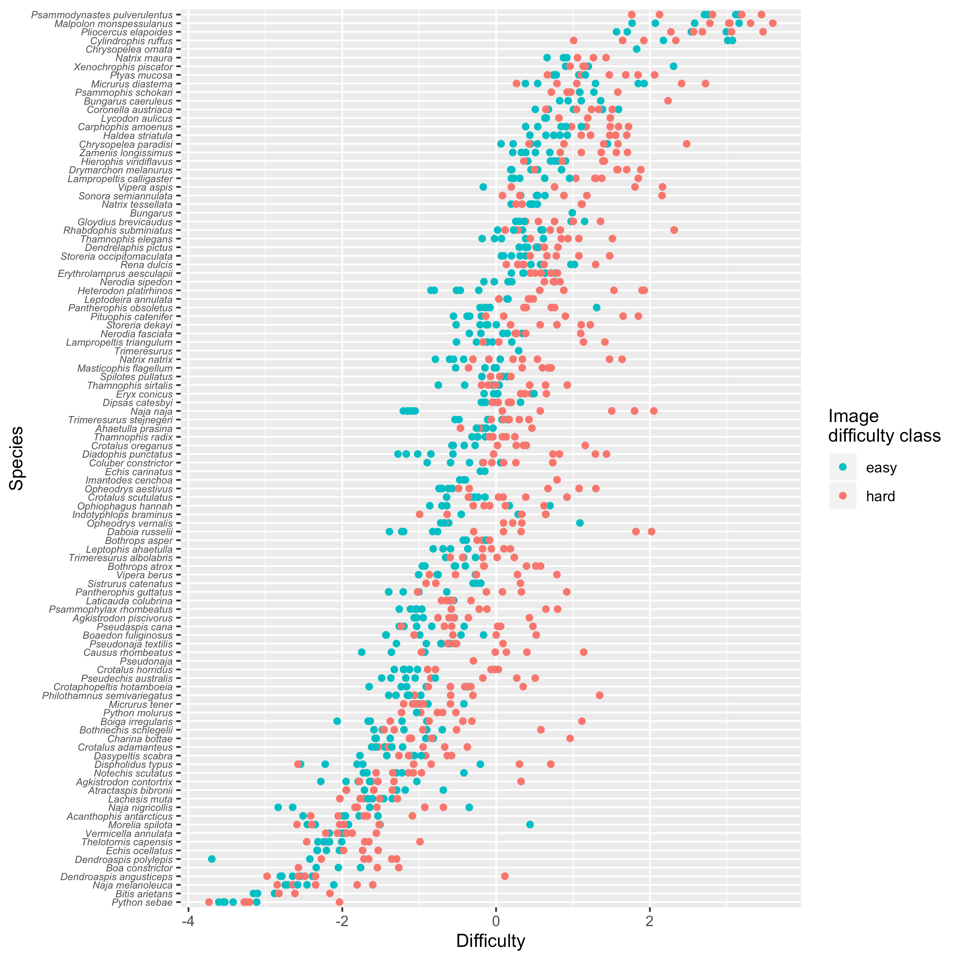

Supplement: Figure S2 [file rsos201273supp3.png]
