# Supplementary material for: Crowdsourcing snake identification with online communities of professional herpetologists and avocational snake enthusiasts
Source: R Soc Open Sci. 2021 Jan 13;8(1):201273. doi: 10.1098/rsos.201273 (PMC7890515; doi:10.1098/rsos.201273)
Supplement: Table S4 [file rsos201273supp7.docx]

Table S4: Comparison of model fit for six candidate models with differing fixed effects structure. LOOIC is the approximate leave-one-out cross validation statistic (Vehtari et al. 2017); lower values indicate a more parsimonious model. *R^2^*_out_ gives Bayesian R-squared for the held-out data using group-level effects for participant ID, image and snake species; *R^2^*_out*_ as above but without using the group-level effects. MEA_out_ and MEA_out*_ give the mean absolute error of predictions for held-out data, with and without group-level predictors respectively. Model formula show the population-level covariates included in each of the candidate models. All models were fitted to the same dataset and with the same group-level effects for the score and discrimination models as described in Appendix A.

| Candidate Model | Δlooic | looic | std err. looic | *R^2^*_out_ | *R^2^*_out*_ | MEA_out_ | MEA_out*_ | Model Formula |
| --- | --- | --- | --- | --- | --- | --- | --- | --- |
| m3 | 0 | 86500.17 | 344.7 | 0.47 | 0.36 | 0.83 | 1.17 | key_family + photo_region +  home_region + taxa_repeat +  photo_region:home_region |
| m1 | -36.35 | 86536.51 | 344.6 | 0.47 | 0.36 | 0.83 | 1.16 | key_family + photo_region +  home_region + taxa_repeat +  image_quality + photo_region:home_region |
| m4 | -277.26 | 86777.43 | 341.61 | 0.47 | 0.36 | 0.84 | 1.17 | key_family + photo_region +  home_region + taxa_repeat |
| m2 | -321.79 | 86821.96 | 341.53 | 0.47 | 0.36 | 0.84 | 1.16 | key_family + photo_region +  home_region + taxa_repeat + image_quality |
| m6 | -3430.65 | 89930.82 | 332.72 | 0.45 | 0.34 | 0.89 | 1.22 | key_family + photo_region +  taxa_repeat |
| m5 | -3459.39 | 89959.56 | 332.73 | 0.45 | 0.34 | 0.89 | 1.21 | key_family + photo_region +  taxa_repeat + image_quality |
