# Supplementary material for: Crowdsourcing snake identification with online communities of professional herpetologists and avocational snake enthusiasts
Source: R Soc Open Sci. 2021 Jan 13;8(1):201273. doi: 10.1098/rsos.201273 (PMC7890515; doi:10.1098/rsos.201273)
Supplement: Appendix A [file rsos201273supp8.docx]

**Appendix A:** Technical summary of the Bayesian Item Response Theory model used in “Crowdsourcing snake identification with online communities of professionals and avocational enthusiasts”

***I. Model Structure***

Let *y_n_* denote the accuracy of each identification provided by participants in the online snake identification challenge. Accuracy is an ordinal variable taking one of four integer values: 1 = incorrect, 2=family correct, 3=genus correct and 4=species correct. Given the ordered and categorical nature of this response variable, we used a variant of Item Response Theory (IRT) known as the graded response model (GRM) in our analysis (Samejima 1997). In the GRM, the probability of each level of accuracy is given by the following (notation following Bürkner (2020)):

$P\left( y_{n}=c \right)= F\left( \tau_{c}- \psi_{n} \right)-F(\tau_{c-1}-\psi_{n})$,

where *y_n_*, is the accuracy of each response *n*, *c* is the level of identification accuracy (1 to 4), $\psi_{n}$ is the linear predictor for response *n*, and $\tau_{c}$is a set of threshold parameters corresponding to the *C* levels of identification accuracy. We modeled the predictor $\psi$ as a non-linear function of two parameters, $\alpha$ and $\mu$, making this a two-parameter logistic or “2PL” model in the terminology of IRT (Bürkner 2020). Specifically,

$\psi_{n}= \alpha_{ik}\left( \mu_{pik} \right),$

$$\mu_{pik}=\theta_{p}- \zeta_{1i}-\eta_{1k}- \sum_{j=1}^{J} b_{j}x_{jik},$$

where $\alpha_{ik}$is the item discrimination (discussed further below) and $\mu_{pik}$ is the expected accuracy of each response. Accuracy depends on the knowledge of each participant, $\theta_{p}$, the difficulty of each image, $\zeta_{1i}$, the difficulty of each species, $\eta_{1k},$ the values of *J* covariates associated with each image and species, $x_{jpi}$, and the fitted regression coefficients, $b_{j}$. The intercepts associated with participant knowledge are assumed to be normally distributed with a standard deviation fixed at one, $\theta_{p}\sim N\left( 0, 1 \right).$ In the commonly used terminology of linear regression modeling, the intercepts for person, image and species are analogous to “random” effects and the $b_{j}$ coefficients analogous to “fixed” effects.

The value of the discrimination parameter, $\alpha_{ik}$, is determined by the following equation,

$$\alpha_{ik}=\exp\left( {1+\zeta}_{2i}+\eta_{2k} \right),$$

where $\zeta_{2i}$ is a measure of the discrimination associated with each individual snake image and $\eta_{2k}$ is a measure of discrimination associated with each species. The sum of the discrimination terms is exponentiated to ensure that the $\alpha_{ik}$ is positive (Bürkner 2020). To account for any correlation between image difficulty and image discrimination, we assume that $\zeta_{1i}$ and $\zeta_{2i}$ are drawn from a multivariate normal distribution with covariance matrix $\Sigma_{\zeta}$, thus $\left( \zeta_{1i},\zeta_{2i} \right)\sim MVN\left( 0, \Sigma_{\zeta} \right).$ Likewise, we assume species difficulties and discriminations are drawn from a multivariate normal distribution as well, $\left( \eta_{1i}, \eta_{2i} \right)\sim MVN\left( 0, \Sigma_{\eta} \right)$.

***II. Bayesian Priors***

Each parameter fitted in the Bayesian GRM requires specifying a prior distribution. We used the weakly informative priors following Bürkner (2020) (Table A1).

**Table A 1 Summary of priors**

| Parameter | Prior Distribution | Interpretation |
| --- | --- | --- |
| $\boldsymbol{b}_{\boldsymbol{j}}$ | $\sim uniform$ | Coefficients for population-level (aka ‘fixed’) effects |
| $\boldsymbol{\tau}_{\boldsymbol{c}}$ | $\sim student t(0, 10)$ | Varying intercepts for each level of accuracy (C = 4) |
| $\boldsymbol{Intercept}_{\boldsymbol{disc}}$ | $\sim normal(0, 1)$ | Intercept for discrimination model |
| $\boldsymbol{L}_{\boldsymbol{2}}\boldsymbol{,}\boldsymbol{L}_{\boldsymbol{3}}$ | $\sim LKJ Cholesky(1)$ | Sets correlation between image (or species) difficulty and image (or species) discrimination |
| $\boldsymbol{\sigma}_{\boldsymbol{disc.}}^{\boldsymbol{2}}$ | $\sim student t(0,10)$ | Standard deviation of discrimination intercept |
| $\boldsymbol{\sigma}_{\boldsymbol{id}}^{\boldsymbol{2}}$ | $\sim constant(1)$ | Standard deviation of person effect |
| $\boldsymbol{\sigma}_{\boldsymbol{1, image}}^{\boldsymbol{2}}$ | $\sim normal(0,1)$ | Standard deviation of image effect on difficulty |
| $\boldsymbol{\sigma}_{\boldsymbol{1,species}}^{\boldsymbol{2}}$ | $\sim normal(0,1)$ | Standard deviation of species effect on difficulty |
| $\boldsymbol{\sigma}_{\boldsymbol{2, image}}^{\boldsymbol{2}}$ | $\sim normal(0,1)$ | Standard deviation of image effect on discrimination |
| $\boldsymbol{\sigma}_{\boldsymbol{2,species}}^{\boldsymbol{2}}$ | $\sim normal(0,1)$ | Standard deviation of species effect on discrimination |

***III. Writing the model formula in R***

We used the `brms` package in the R statistical language to fit the model as specified above (R Core Team 2015, Bürkner 2017). The brms package provides a high-level interface for writing models that are run by the Stan modeling language (Stan Development Team 2015a). Following examples for a 2PL GRM provided in Bürkner (2020), and making use of R’s linear regression modeling syntax, the formulae for the most complex model was as follows:

R> y ~ family + region + home + taxa_repeat + quality + home:region + (1|i|item) + (1|k|species) + (1|id)

R> disc ~ 1 + (1|i|item) + (1|k|species)

In the example above, the first line of code is the formula for the average accuracy, the second is the formula for the discrimination parameter. Terms in parenthesis denote hierarchical error models (aka ‘random’ effects). Terms (1|i|item) and (1|k|species) included in both formulae indicate that these effects are expected to be correlated (Bürkner 2020). Terms ‘family’, ‘region’, etc. correspond to fixed effects as described in the main text. Data and code required to replicate analyses are available on github: www.github.com/akleinhesselink/snapp.

***IV. Sampling Diagnostics***

Each model was sampled using the No-U-Turn Sampling (NUTS) algorithm in the Stan modeling language (Stan Development Team 2015a, Bürkner 2017). We ran 4000 iterations with a 2000 iteration burn-in period on four independent sampling chains. We assessed sampling convergence visually by plotting the posterior chains for sampled parameters (Figure A1). This revealed that chains converged and were generally well mixed.


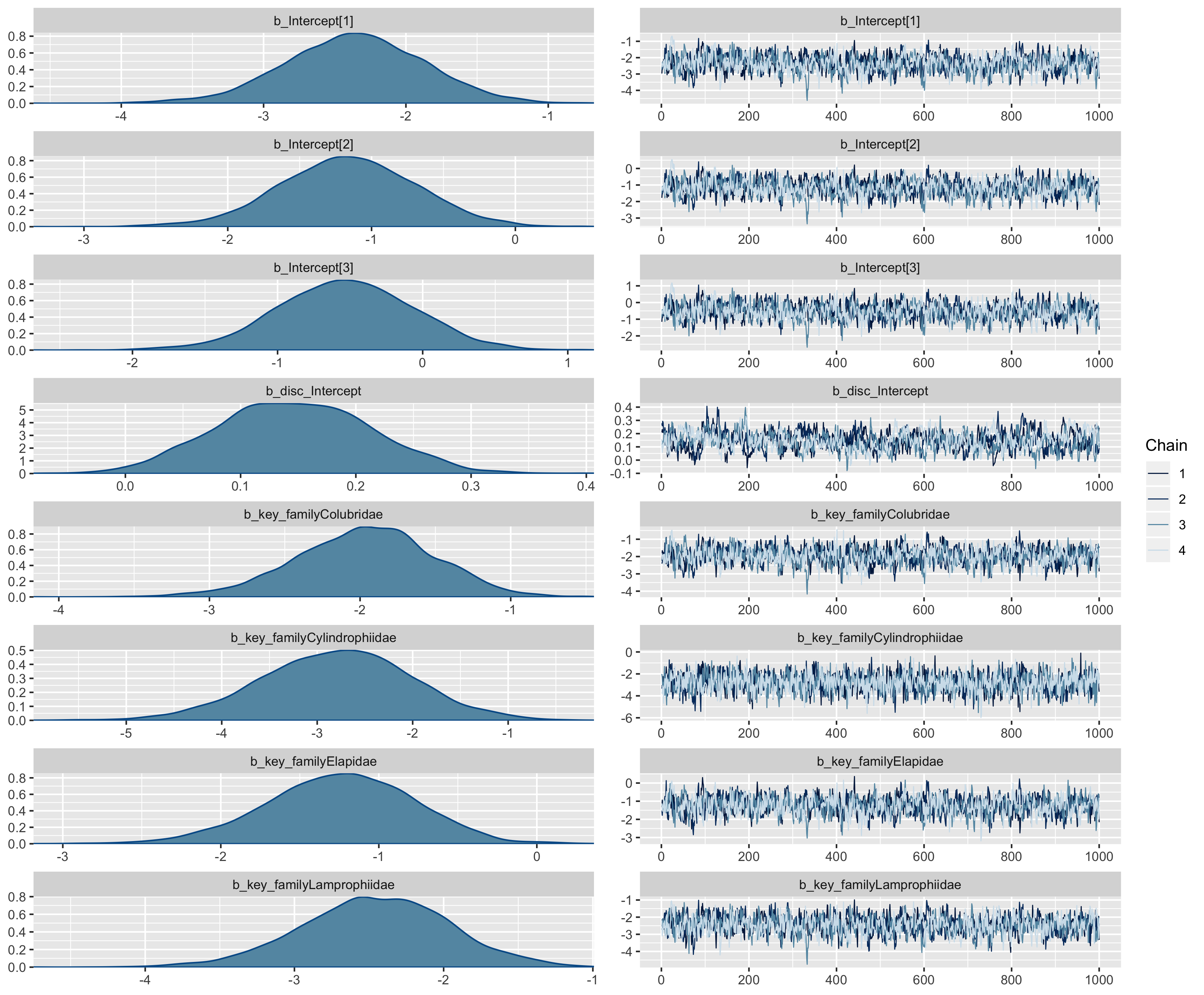


**Figure A1 (part 1). Posterior distributions (left) and traceplots for NUTS sampling chains (right) for the first 8 parameters in the IRT model used in the manuscript. Plot continues below.**


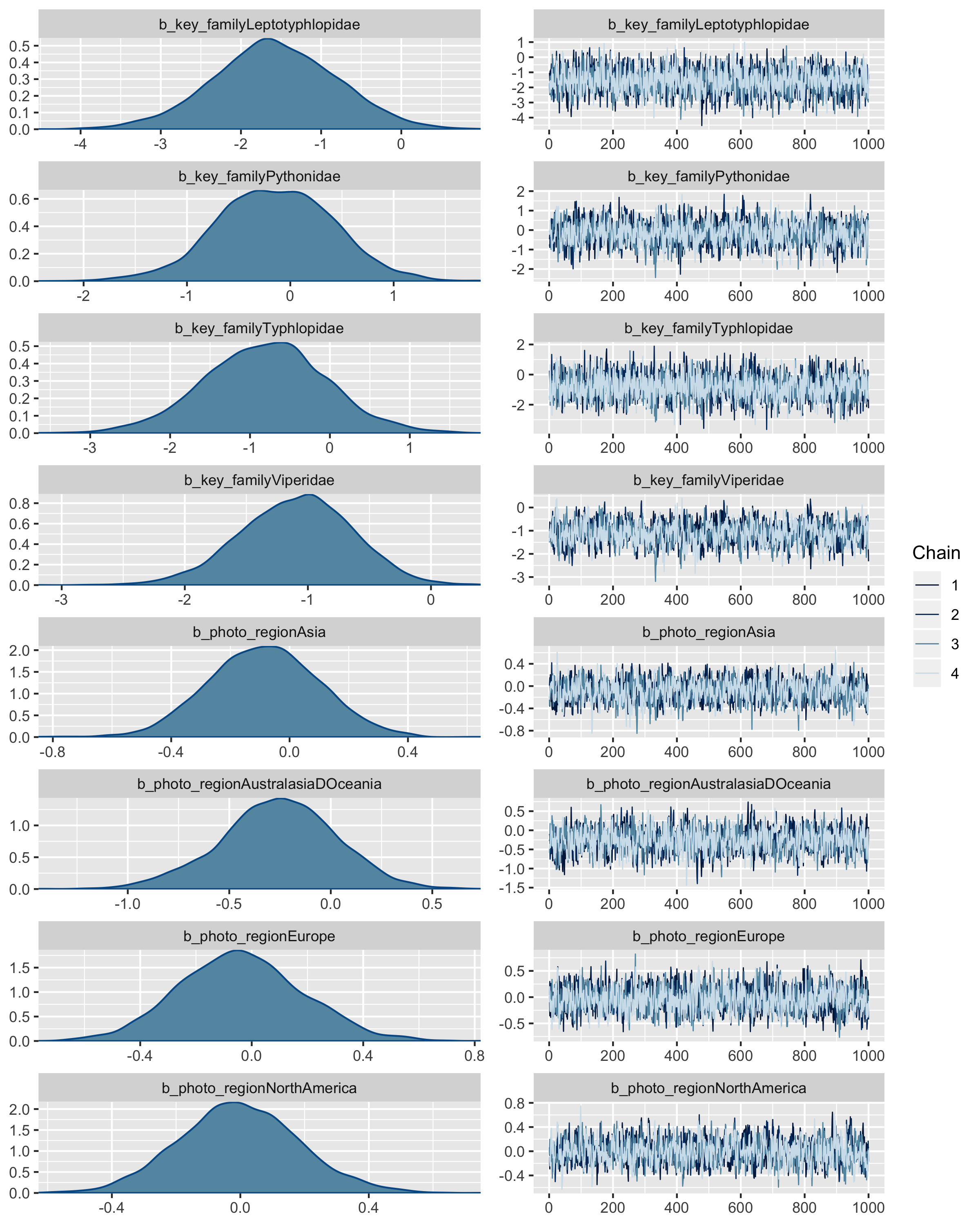


**Figure A1 (part 2). Posterior distributions (left) and traceplots for NUTS sampling chains (right) for 8 parameters in the IRT model used in the manuscript. Plot continues below.**

**
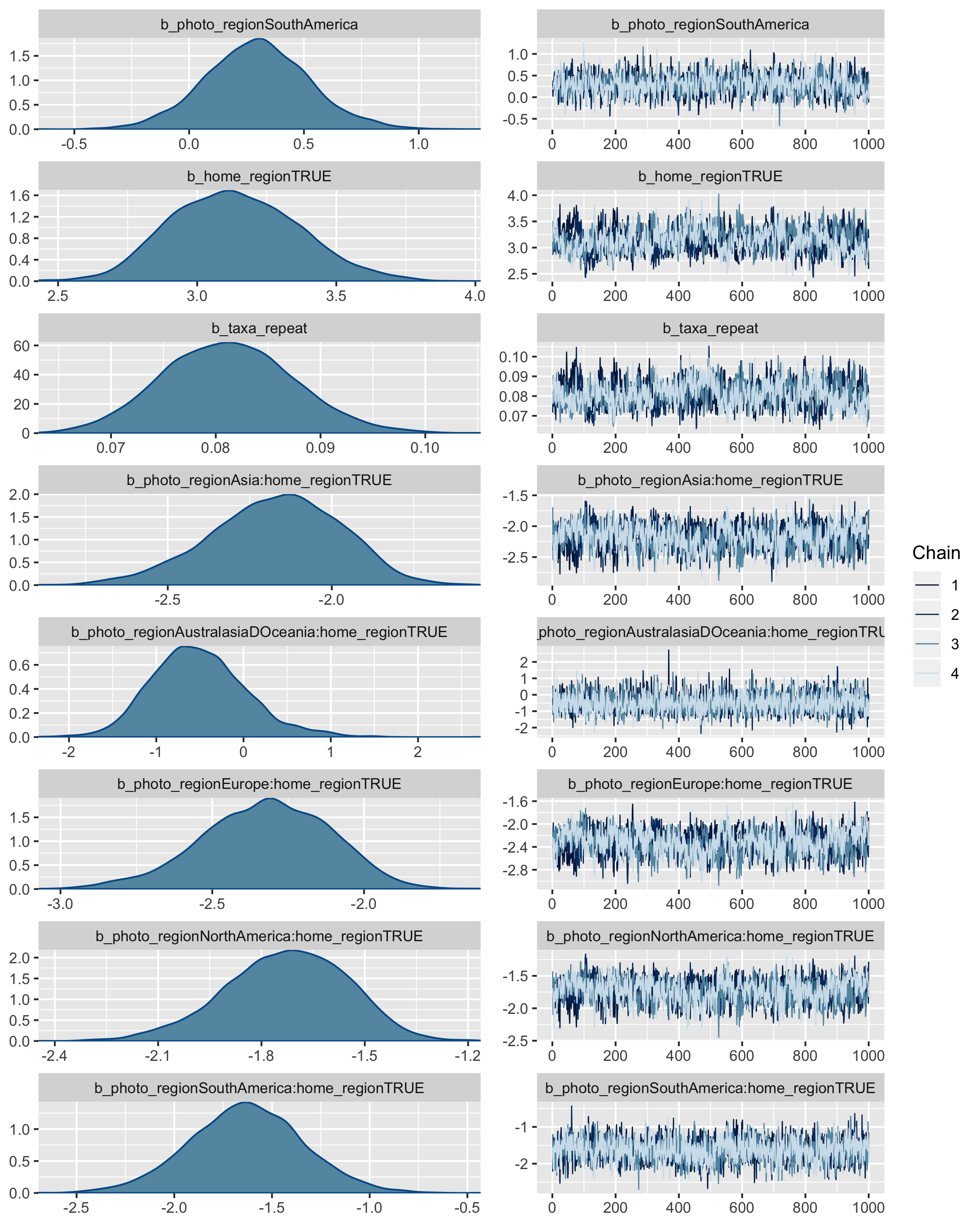
**

**Figure A1 (part 3). Posterior distributions (left) and traceplots for NUTS sampling chains (right) for 8 parameters in the IRT model used in the manuscript. Plot continues below.**


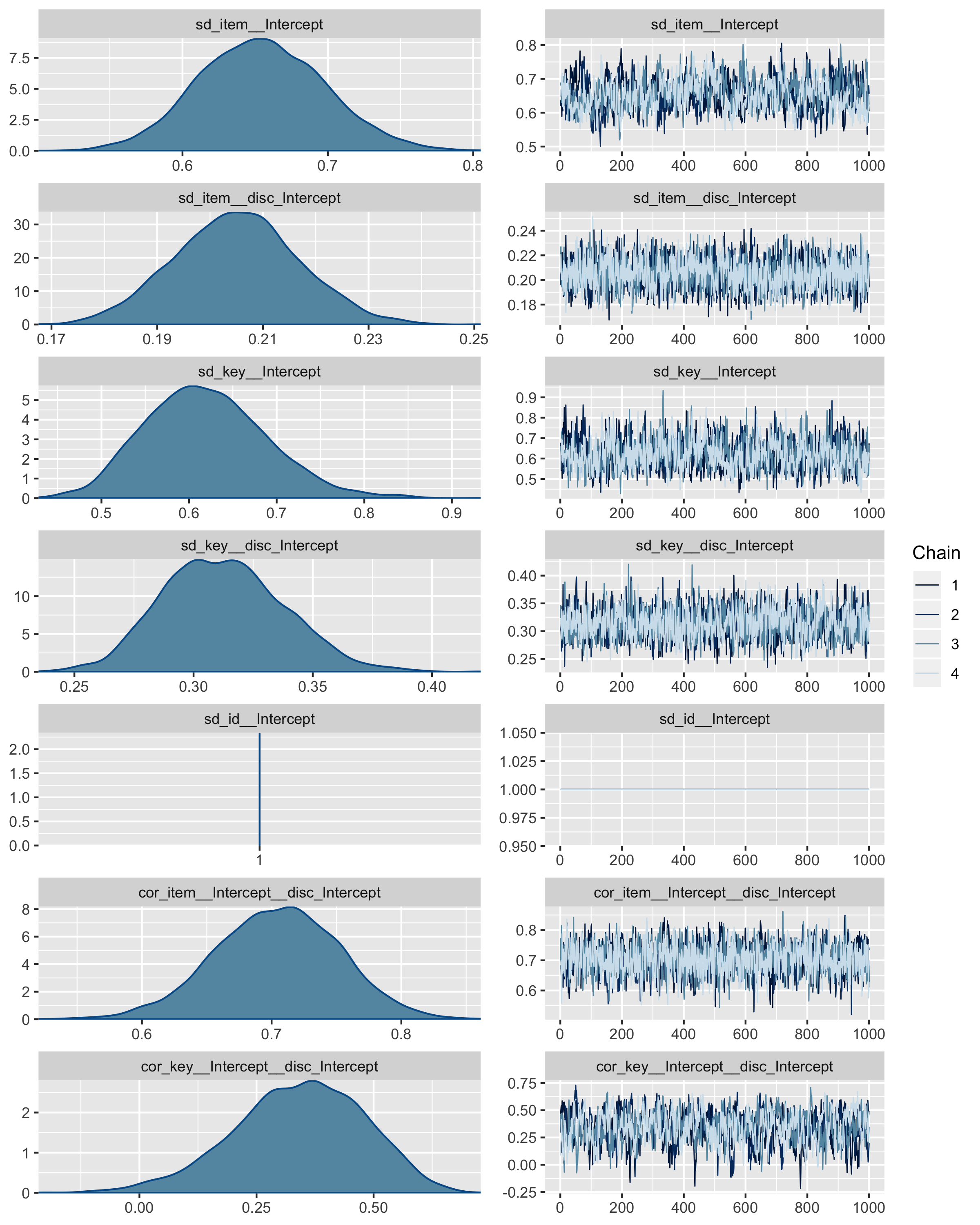


**Figure A1 (part 4). Posterior distributions (left) and traceplots for NUTS sampling chains (right) for 8 parameters in the IRT model used in the manuscript. The standard deviation for “id” (person) was fixed at 1 which is standard practice in IRT (Bürkner 2020).**

In addition, we assessed effective sample size (ESS) for each parameter (Table S1). Tail ESS for all parameters were above the recommended threshold of *n* = 400, but for three parameters bulk ESS was slightly below this (for $\sigma_{image}^{2}$, $Intercept_{disc}$, $b_{home})$. For all parameters the R-hat split chain statistic was less than 1.05 indicating good convergence (Stan Development Team 2015b).

***V. Model Fit***

We used posterior predictive checks to compare predictions generated from the posterior distribution of fitted models to the observed data (Stan Development Team 2015b, Bürkner 2017). Before fitting the models, we split data into a training and testing set, with the testing set held out from model fitting. Here we show posterior predictive checks for both in sample (Figure A2) and out of sample data (Figure A3).


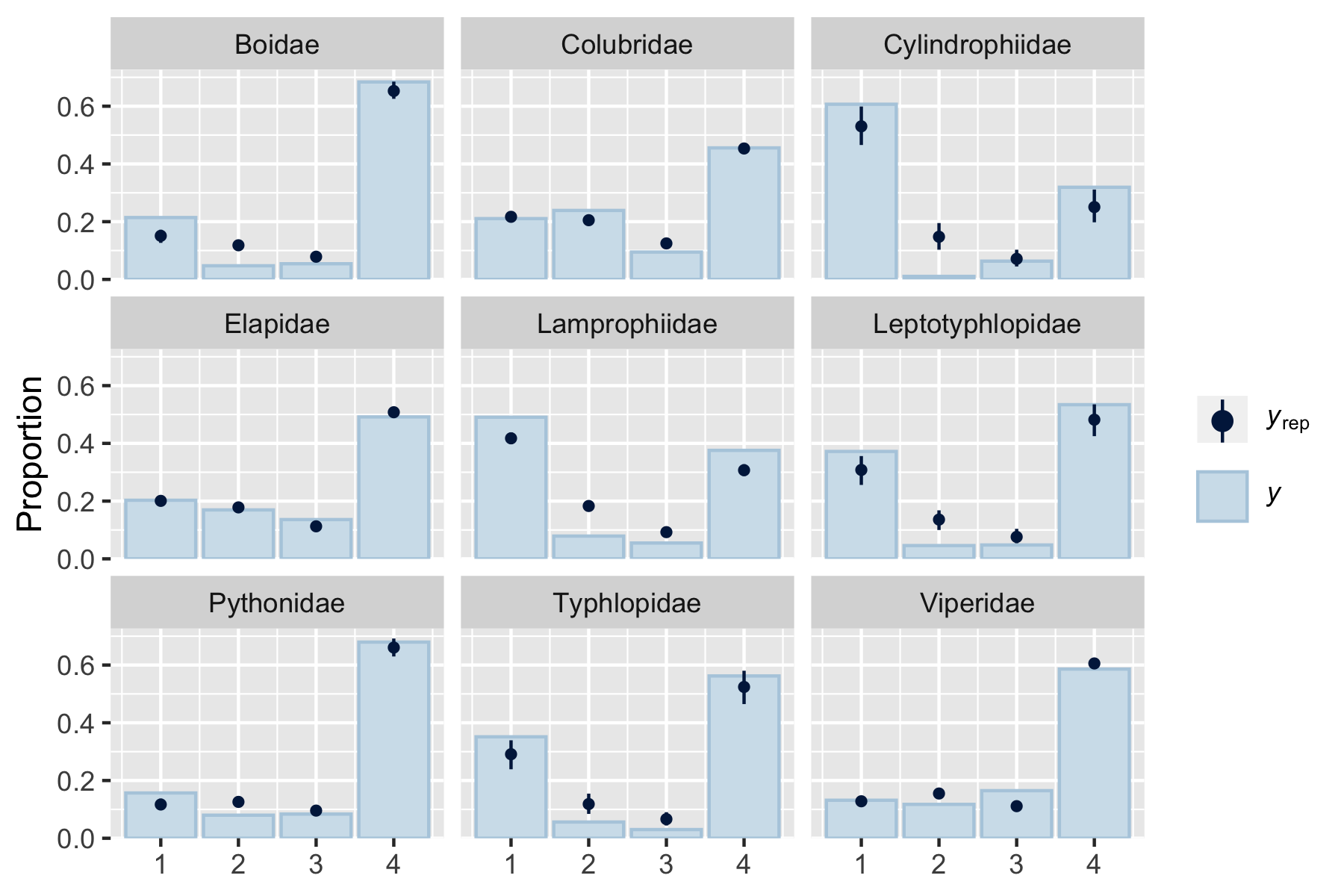


**Figure A2. Graphical posterior predictive check for in-sample data. Proportion of observed accuracy scores for each family are shown with the bars. Proportion of predicted accuracy scores generated from the model posterior are shown with circles and 95% Bayesian credible intervals.**


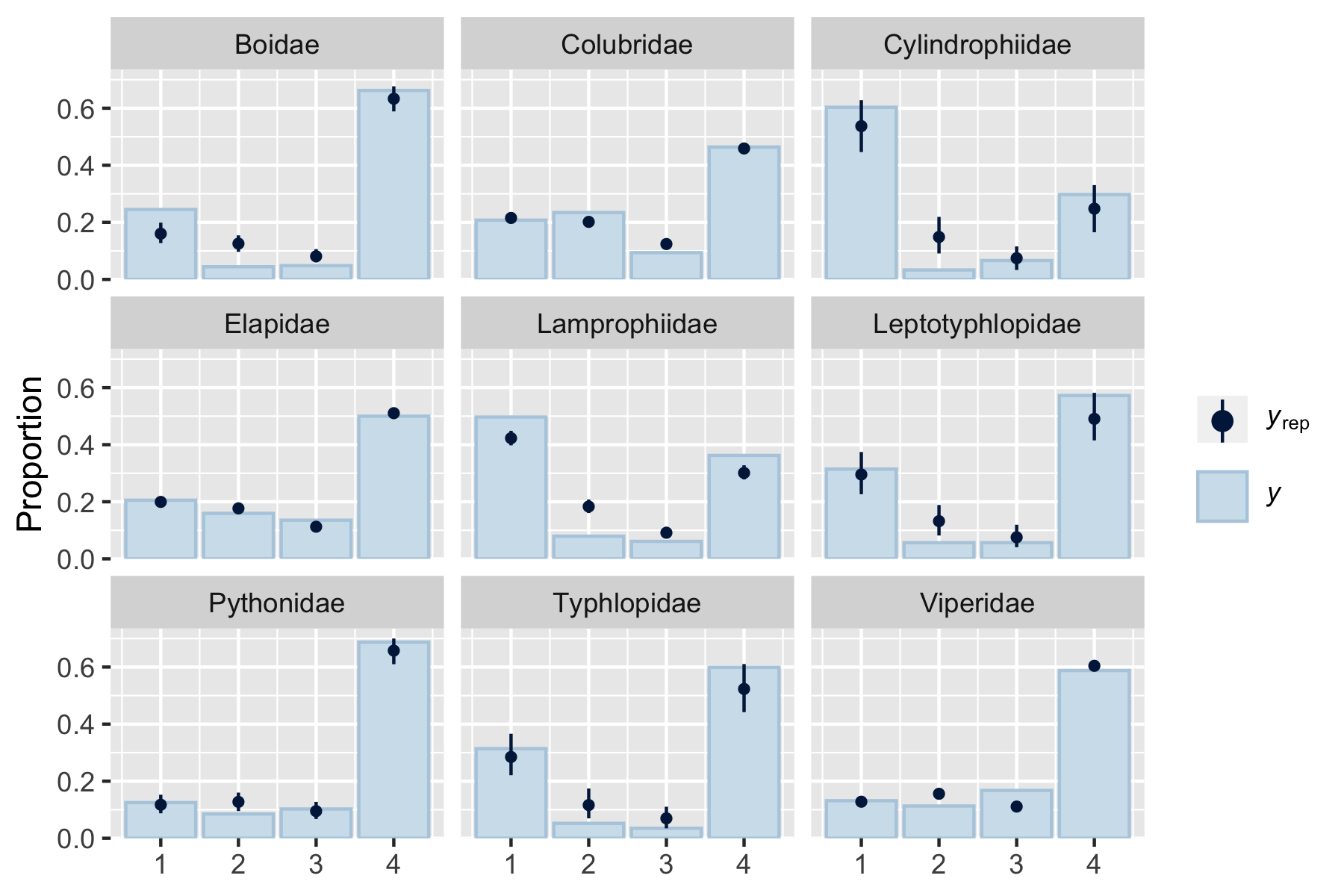


**Figure A3. Graphical posterior predictive check for out-of-sample data. Proportion of observed accuracy scores for each family are shown with the bars. Proportion of predicted accuracy scores generated from the model posterior are shown with circles and 95% Bayesian credible intervals.**

The pattern of expected scores for each family show good alignment with the pattern of observed scores for both in-sample and out of sample data (Figs A2, A3). However, the model appears to be underestimating the error associated with score categories as in many cases the 95% credible intervals do not contain the observed proportions.

**References cited in Appendix A:**

Bürkner, P.-C. 2017. Advanced Bayesian Multilevel Modeling with the R Package brms. arXiv:1705.11123 [stat].

Bürkner, P.-C. 2020. Bayesian Item Response Modeling in R with brms and Stan. arXiv:1905.09501 [stat].

R Core Team. 2015. R: A Language and Environment for Statistical Computing. R Foundation for Statistical Computing, Vienna, Austria.

Samejima, F. 1997. Graded Response Model. Pages 85–100 *in* W. J. van der Linden and R. K. Hambleton, editors. Handbook of Modern Item Response Theory. Springer, New York, NY.

Stan Development Team. 2015a. Stan: A C++ Library for Probability and Sampling. Stan Development Team.

Stan Development Team. 2015b. Stan Modeling Language User’s Guide and Reference Manual, Version 2.10.0. Stan Development Team.
